# Historical Aspects of Herbal Use and Comparison of Current Regulations of Herbal Products between Mexico, Canada and the United States of America

**DOI:** 10.3390/ijerph192315690

**Published:** 2022-11-25

**Authors:** Patricia Rojas, Helgi Jung-Cook, Elizabeth Ruiz-Sánchez, Irma Susana Rojas-Tomé, Carolina Rojas, Arely M. López-Ramírez, Aldo Arturo Reséndiz-Albor

**Affiliations:** 1 Laboratorio de Inmunidad de Mucosas, Sección de Estudios de Posgrado e Investigación, Escuela Superior de Medicina del Instituto Politécnico Nacional, Plan de San Luis esq. Salvador Díaz Mirón s/n, Mexico City C.P. 11340, Mexico; 2 Facultad de Química, Universidad Nacional Autónoma de México, Mexico City C.P. 04510, Mexico; 3 Laboratorio de Neurotoxicología, Instituto Nacional de Neurología y Neurocirugía “Manuel Velasco Suárez”, SSA, Av. Insurgentes Sur No. 3877, Mexico City C.P. 14269, Mexico; 4 Laboratorio de Neuropsicofarmacología, Instituto Nacional de Neurología y Neurocirugía “Manuel Velasco Suárez”, SSA, Av. Insurgentes Sur No. 3877, Mexico City C.P. 14269, Mexico; 5 Instituto de Investigaciones Biomédicas, Universidad Nacional Autónoma de México, Mexico City C.P. 04510, Mexico

**Keywords:** herbal products, legislation for herbal products, regulations for herbal products, regulatory science, herbal medicine, botanical drug, dietary supplements, natural health products, Mexico, Canada, United States of America

## Abstract

Increased life expectancy and high costs of medicines and medical care have led to the use of herbal products. However, these items may contain toxic compounds that have an impact on public health. We will focus on the regulatory aspects and differences of these products marketed in the North American region (USA-Mexico-Canada) from government websites and selected literature. Mexico has an ancestral tradition of using plants for the treatment, improvement, and maintenance of human health as compared with Canada and the USA Currently, the use of herbal products in this region has a regulatory framework. The legal framework in these three countries is related to their history, idiosyncrasies, socio-economic and cultural aspects. Therefore, there are different public policies for herbal products consumed in the region. Mexico has a more specific classification of these products. In Canada, all herbal products are classified as natural health products and the safety and efficacy must be scientifically proven. In the USA, the development of botanical drugs is very recent. In particular, both herbal products classified as food supplements in Mexico and dietary supplements in the USA may have risks in both safety and efficacy.

## 1. Introduction

Medicinal plants have been used since ancient times by many people worldwide. Nowadays, plants are often used for the development of various products that are useful for human health, including medicines, because they contain specific compounds with biological activity (e.g., alkaloids and terpenoids) and which have been used empirically in traditional medicine [1,2].

In this regard, the United Nations Declaration on the Rights of Indigenous Peoples is a document of relevance for the inclusion and rights of the world’s Indigenous people, including the maintenance of their traditional medical practices [1]. It obliges United Nations member states in partnership with Indigenous people to develop policies under national laws and programmes for their protection. In particular, article 24 of the Declaration on Health states “indigenous peoples have the right to their traditional medicines and health practices. The plants, animals and minerals used in medicines shall be protected” (UN document A/RES/61/295) [1]. Likewise, the World Health Organization (WHO), in the Beijing Declaration, recognises traditional medicine as a relevant resource in primary health care [2].

Additionally, the World Trade Organization has highlighted the contribution of traditional medicine to the health of many communities [3]. However, the benefits of this type of medicine have been underestimated in many developed countries, but successfully used for the prevention and management of chronic diseases associated with lifestyle [4]. In addition, increased life expectancy, rising health care expectations, high health care costs, and rising drug costs, among others, have prompted many countries to reconsider their interest in traditional medicine. Traditional medicine has a long history representing the skills, knowledge, and practices based on beliefs, theories, and experiences of Indigenous people and is used for the prevention, diagnosis, improvement, or treatment of mental or physical illness and general health maintenance [4]. 

It is important to mention that according to a WHO report, the Region of the Americas has developed national policies, laws, regulations, and programmes, as well as established traditional medicine offices [4]. The purpose is to generate public policies for the recognition and safe use of traditional medicine as well as herbal products. In this perspective, the use of herbal products in human health has markedly increased in Western countries in the last decade, mainly in developed countries [5].

The plants have very well-known medicinal uses and have been the basis for the development of multiple pharmaceutical drugs for the treatment of various diseases with worldwide relevance, such as the symptomatic treatment of dementia [6] and diabetes [7]. In particular, pharmacopoeias precisely detail the procedures for identifying and assessing the quality of plants used in the production of herbal drugs.

Although not all herbal products for human health that are sold to the public by different market systems are safe, efforts are made to regulate the quality control and safety. Thus, it is important to have a regulatory status for herbal medicines. In this review, we will focus on the regulatory aspects of herbal products marketed in the North American region under the United States-Mexico-Canada Trade Agreement (USMCA). We will emphasise that regulation is based on their history, idiosyncrasies, and cultural aspects. 

This work highlights the importance for each region to have appropriate legislation and regulation to help ensure the quality and safety of herbal products on the market to improve the protection of human health.

## 2. Materials and Methods

This review was conducted using electronic databases of the WHO, government regulations, and policies from the official websites of Mexico, Canada, and the USA, both in English and Spanish. Furthermore, selected references from PubMed, MEDLINE, and Google Scholar databases were included. The key terms used to find relevant information included: herbal product regulations, herbal medicines regulations, traditional medicine, Indian herbology of North America, medicinal plants, culture of medicinal plants, Mesoamerica, Indian tribes in USA, American Indians, Indigenous peoples in the United States, Indian tribes in Canada, Canada’s First Nations, Biblioteca Digital de la Medicina Tradicional Mexicana, plantas medicinales de México. 

## 3. WHO and Traditional Medicine

The WHO has reported significant growth in the use of traditional and complementary medicine (T&CM) in 170 member states (88%) [4], where it is frequently used for the prevention and management of chronic diseases associated with today’s lifestyle, but is often under-recognised. 

The WHO defines “traditional medicine as the sum total of the knowledge, skills and practices based on the theories, beliefs and experiences indigenous to different cultures, used in the maintenance of health as well as in the prevention, diagnosis, improvement or treatment of physical and mental illness” [4]. 

On the other hand, there are other widely used terms such as “complementary medicine” and “alternative medicine”, which are an extensive group of health care practices that do not belong to traditional medicine and are not completely incorporated into the prevailing global health care system, although the terms mentioned above have the same meaning in some countries. Thus, over the last two decades, the growth of T&CM is becoming an alternative to cover health service needs, with the use of plants being a relevant part of these practices. In particular, 124 member states have legislation and/or regulations on plant-based medicine. The WHO has established the following definitions [4]: (a) “conventional pharmaceuticals are medicinal drugs used in conventional systems of medicine with the intention to treat or prevent disease, or to restore, correct or modify physiological function”; (b) “herbal medicines include herbs, herbal materials, herbal preparations and finished herbal products that contain, as active ingredients, parts of plants, other plant materials or combinations thereof. In some countries, herbal medicines may contain by tradition, natural organic or inorganic active ingredients that are not of plant origin (e.g., animal and mineral material)”.

In particular, India, China, and Mexico have integrated medicinal plants, an ancient wisdom, into their national health policies [4].

## 4. Mexico

### 4.1. Traditional Medicine in Mexican Cultures

Mexico has a long tradition in the use of medicinal plants [8,9] and is the country with the second largest number of registered medicinal plants after China [10]. 

The development of Indigenous traditional medicine in the cultures settled in Mesoamerica dates back to 1500 B.C., starting with the mother culture, the Olmec, and continuing with the Toltecs, Teotihuacans, and Mayans [11]. However, this ended with the fall and conquest of the Aztec empire in 1521 by Spanish colonisers, who destroyed much information on the use of plants for medicinal purposes. Nonetheless, recovery of the medical history of the Aztecs was achieved after the conquest. Thus, in 1552, the first book of medicinal plants of the Indians on the American continent was compiled and is known as the *de la Cruz-Badiano Codex* (Código de la Cruz-Badiano), written by the Nahuatl Indian Martin de la Cruz and translated by Juan Badiano [12]. Aztec medical theory was based on religion, astronomy, divination, and the polarity of cold and warm.

Currently, the Indigenous population of Mexico is estimated to be around 25.5 million (~21.5%) [13]. These Indigenous communities are the main practitioners of herbal medicine in curing common illnesses [14].

### 4.2. Mexican Botanical Diversity and Medicinal Plants

Mexico has a great botanical richness due to the variety of climates from tropical to desert environments, resulting in a variety of microclimates and ecosystems. The National Commission for the Knowledge and Use of Biodiversity (CONABIO) estimates that more than 4000 plant species have medicinal properties, representing around 15% of the country’s flora and ranking second in the world. The Mexican Social Security Institute (IMSS) has documented 2000 medicinal plant species [15]. The WHO estimates that 90% of the Mexican population uses or has used medicinal plants. According to the Ministry of Environment and Natural Resources (SEMARNAT), 5% of plants for medicinal purposes have been scientifically studied [16]. However, overexploitation of medicinal plants for commercial purposes can lead to the loss of ecosystems; therefore, the development of propagation and production protocols is important. Mexico has made efforts to have the traditional knowledge of Mexican medicinal plants regularised in the legal framework for the production and commercialisation of herbal remedies and medicines, as well as food supplements.

In Mexico, there is an atlas of traditional Mexican medicinal plants that includes 1045 monographs of the most frequently used medicinal plants [17]. It describes various aspects including, among others, botanical description, experimental information, and field information from traditional practitioners.

### 4.3. Mexican Legal and Regulatory Framework of Herbal Products

Mexico recognised traditional medicine in the General Health Law, which includes a specific national programme (Table 1) [18]. The Federal Commission for the Protection against Health Risks (COFEPRIS) is a decentralised organisation of the Ministry of Health in charge of the control, regulation, and promotion of health [19] as stipulated in the General Health Law [18] and other legal provisions. It regulates the right to health protection of products intended for human consumption, including those containing raw plants material.

COFEPRIS is composed of [19]: (1) Head in charge of coordinating the activities of all commissions; (2) Commissioner for Health Promotion in charge of information on special electronic prescriptions, dispensing of medicines, citizen contact network, proactive transparency, and good health practice guides in pharmacies with adjacent clinics; (3) Commissioner for Sanitary Operation who maintains the sanitary control of products, services, activities, and establishments; (4) Commissioner for analytical control and coverage extension which generates reliable and timely analytical results, resolutions, and technical opinions for decision-making to protect the health of the Mexican population; (5) Health Authorisation Commission, responsible for issuing licenses, registration, and certificates; (6) Evidence and Risk Management Commission provides information on food additives, climate change and health, genetically modified organisms, environmental health, water quality, clean beaches, pharmacovigilance, pharmacopoeia, and technovigilance.

In general, herbal products in Mexico can be classified as non-prescription, prescription, general food products, food supplements, functional foods, and health foods. They are sold as herbal medicines, as well as health and nutritional products [18]. In addition, a national regulation for herbal medicines has been included in the Herbal Pharmacopoeia of the United Mexican States (FHEUM) since 2001, when the first edition was published.

In particular, Mexico has three pharmacopoeias: (1) general pharmacopoeia; (2) the homeopathic pharmacopoeia; and (3) the herbal pharmacopoeia.

#### Classification of Herbal Products in Mexico for Use and Marketing

In Mexico, plant products for human consumption are mainly classified as herbal medicines, herbal remedies, and food supplements.

**Herbal medicine.** Article 224 section B III of the General Health Law states that “an herbal medicine is one that is produced from plant material or any derivative thereof, where the main ingredient may be the underground or aerial part of the plant” whose therapeutic efficacy and safety have been scientifically confirmed in the national or international literature [18]. This definition includes extracts, tinctures, fatty and essential oils, resins, and juices of plants that are presented in pharmaceutical form. Excipients and additives may be added to the formulation. An isolated or injectable active ingredient is not considered an herbal medicinal product.

These types of medicines must comply with a legal framework and other considerations that are described in articles 221 (section I), 222, 223, 224 section B III, and 225 of the General Health Law [18], Health Supplies Regulation articles 66, 67, and 68, [20] and the Mexican Official Standard NOM-072-SSA1-2012 [21] (labelling of herbal medicines and herbal remedies).

According to articles 66, 67, and 68 of the Health Supplies Regulation, herbal medicines cannot include psychotropic or narcotic substances, nor mixtures with other banned substances such as ephedrine [20].

In order to obtain registration, good manufacturing practices of the medicinal product must be complied with, as well as the certification of the active ingredients. This process is verified by COFEPRIS. The agency considers the quality requirements, standards, and technical bases for pharmaceutical raw materials established in the Herbal Pharmacopoeia of the United Mexican States. This document issued by the Mexican Ministry of Health contains the general methods for analysing the purity, quality, and identity of herbal medicinal products permitted for use in the country. To be marketed, these products must be identified distinctively. The registration of herbal medicinal products must contain the expiry date, dosage and route of administration, adverse reactions, therapeutic indication, and alpha-numeric registration code, among other characteristics.

**Herbal remedy.** Article 88 of the Health Supplies Regulation considers that an “herbal remedy is obtained from medicinal plants and their parts, individually or in combination, and their derivatives, and presented in pharmaceutical form. It is used for the relief of symptoms of a disease by traditional or folk knowledge. It shall not contain psychotropic or narcotic substances, allopathic drugs or other substance(s) which by their concentrations represent a health risk”. The plants used as raw material must be free of microbial flora, and microbiological contamination must be avoided in their manufacture. These products must follow the requirements of the Health Supplies Regulation (articles 88 to 98) [19], the Official Mexican Standards NOM-248-SSA1-2011 (good manufacturing practices for establishments dedicated to the manufacture of herbal remedies) [22], and NOM-072-SSA1-2012 (labelling of herbal medicines and herbal remedies) [21]. Additionally, a health registration authorisation is required, which includes, among other requirements, notice of operation of the factory or laboratory, certificate of microbiological analysis, absence of toxic residues, and specification of the ingredients or formula. It will be assigned an alphanumeric code that must be contained in the package. The sale and supply of these products to the public cannot be advertised as curative. Establishments for the manufacture, distribution, and marketing of herbal remedies are subject to health control and surveillance.

**Food supplement.** The General Health Law in articles 215 (fraction V, VI) and 216 [18] relates to foods and non-alcoholic beverages used to supplement or supplement some components of the total diet, as well as to increase total intake. These products do not require health registration but must comply with the Official Mexican Standards NOM-251-SSA1-2009 (good hygienic practices for the processing of food, beverages, or food supplements) [23]. In addition, articles 168 to 179 of the Regulation on Health Control of Products and Services [24] determine the ingredients that may constitute a food supplement (plant extracts, herbs, dehydrated or concentrated fruits, dehydrated traditional foods in isolation or in combination, fatty acids, metabolites, proteins, carbohydrates, amino acids, and may or may not be added with vitamins or minerals). Substances with recognised pharmacological action or substances with recognised therapeutic, preventive, or rehabilitative properties must not be incorporated. The inclusion of plants with recognised toxicity as described in the Herbal Pharmacopoeia of the United Mexican States is also not permitted. The raw material, in particular dehydrated plants, must be subjected to treatments, controls, and procedures to eliminate microbial flora and chemical or physical residues that may be harmful to health. Such products may be presented in pharmaceutical form and must be labelled on the front, with simple, clear, rapid, and truthful warnings about the maximum levels of energy content, fat, saturated fat, sodium, added sugars, and dietary components that may be risk factors for chronic non-communicable diseases, as well as additional information to be applied by the Ministry of Health. In addition, these retail products must contain the legend “this product is not a medicinal product”.

In Mexico, homeopathic medicines are not classified as herbal medicines because their definition refers to any substance or mixture of synthetic substances or substances of natural origin with rehabilitative, therapeutic, or preventive effects elaborated with the procedures of the Homeopathic Pharmacopoeia of the United Mexican States or those included in other national and international scientific literature [18].

## 5. Canada

### 5.1. Canadian First Nations and Traditional Medicine

Currently about 1.7 million Canadians (~4.9%) identify themselves as Indigenous [25]. In Canada, First Nations are the Indigenous people who occupied lands and were integrated into different nations before the arrival of the European colonisers, including the Inuit and Metis [26].

The traditional medicine of Canada’s Native peoples has been at risk of being lost because it was historically displaced by the current medical system and lost the confidence of the people due to conquest. Thus, traditional methods of healing were suppressed over time by Euro-Canadian missionaries, health professionals, and governments [26]. For example, the surveillance and control of the lives of Native peoples was intensified and institutionalised by federal Indian policy through the Indian Act (1876) [27,28]. In the 1960s, children from these communities were forcibly removed from their families or communities and placed in foster care for the purpose of adoption by non-Native families in Canada. This was due to the idea that the parents of these children did not have appropriate homes and education to provide for them [26], causing intergenerational trauma [29]. Therefore, it has been difficult for the original population to pass on their ancestral wisdom about traditional medicine. Nevertheless, in recent decades, this oppression and cultural marginalisation has been reversing.

The traditional medicinal plant knowledge of Canada’s Indigenous peoples has been passed down orally through the generations and has been of great relevance to their well-being and survival for thousands of years. In particular, the boreal forest areas of Canada have been important in this regard for Indigenous peoples such as the Metis, Cree, Dene, Sekani, Innu, Ojibwa, Chippewa, Abekani, and others [30].

Although the preservation and use of their medicinal plants is recent, several authors have made efforts to compile the medicinal culture of Canada’s Indigenous people. For example, the use of 400 medicinal plants of Eastern Canada’s Indigenous people has been reported [30]. The traditional use of about 546 medicinal plants has been described from Indigenous peoples of the Canadian boreal forest (Metis, Cree, and Dene of Alberta, Manitoba, and Saskatchewan) [30].

### 5.2. Canadian Legal Regulatory Framework of Natural Health Products

Canada has a national policy and national program on T&CM, but regional implementation is the responsibility of the provinces and territories (Table 1). However, the policy of the Natural Health Products (NHPs), also known as traditional remedies or complementary medicines, which include herbal medicines, started in this country in 1999 with the creation of the Office of Natural Health Products. These regulations came into force in 2004 after annexing the NHPs products in subsection 30(1) of the Food and Drugs Act. Therefore, these products are subject to the Food and Drugs Act and Regulations [31,32].

In Canada, herbal medicines, dietary supplements, food supplements, and herbal remedies are considered NHPs and are governed according to Natural Health Products Regulations and therefore are required to demonstrate safety and efficacy to obtain their license before being put on the market [32].

The Health Products and Food Branch of Health Canada is the authority which ensures NHPs are effective, safe, and of high quality according to the Canadian Food and Drugs Act, with the purpose that all products made in Canada or entering the country comply with the requirements [31]. The administration of the regulations of these products is through Health Canada’s Natural Health Products Program, which is made up of three directorates with different responsibilities. (1) The Natural and Non-prescription Health Products Directorate (NNHPD) is the main directorate and is the authority in charge of regulating NHPs, as well as evaluating and issuing licenses for these products. (2) The Marketed Health Products Directorate (MHPD) performs post-approval surveillance, safety, and risk communication of health products, and adverse reaction management of health products including NHPs. (3) The Health Products and Food Branch Inspectorate (HPFBI) is in charge of enforcing the regulations and required enforcement actions. Both consumer and industry complaints or concerns are dealt with by HPFBI [33]. For better functioning, Health Canada periodically consults two external advisory committees: (1) the Expert Advisory Committee (advises on efficacy, safety, and quality of NHPs); and (2) the Management Advisory Committee (advises on the management of NHPs).

All NHPs must be licensed (NHPR, Part 1 Product Licenses, sections 4 and 5) before being offered for sale in Canada [32]. This process requires detailed and accurate product information such as ingredients, dosage, potency, and recommended uses. When a product is licensed, an 8-digit Natural Product Number (NPN) or an 8-digit Homeopathic Medicines Number (DIN-HM) will be obtained, which must appear on the label in order to be marketed (NHPR, Part 1 Product Licenses, section 8, Product number) [32].

Evidence of safety and efficacy of NHPs must be scientifically supported (NHPR, Part 1 Product Licenses, section 5) by information obtained from journals, pharmacopoeias, traditional resources, and clinical trial data. This is in accordance with health claim requirements. The NHPs must be specifically marked including product name, route of administration, dose, product license number, recommended use, complete ingredients, and information on possible adverse reactions (NHPR Part 1, Product Licenses, License Content, section 14) [32]. Furthermore, the site of licensing is required to demonstrate that good manufacturing practices were applied to ensure product quality control and risk management (NHPR, Part 3, Good Manufacturing Practices, section 43–62) [32]. The NHPs are registered in the Licensed Natural Health Products Database.

Canada does not have a pharmacopoeia for herbal medicines. However, the Natural and Non-Prescription Health Products Directorate (NNHPD) Compendium of Monographs (herbals, minerals, vitamins, etc.), the European Pharmacopoeia, The United States Pharmacopoeia, the British Pharmacopoeia, The Pharmacopoeia of the People´s Republic of China, and the Ayurvedic Pharmacopoeia of India are used, although they are not legally binding.

#### Denomination of Herbal Products in Canada for Use and Marketing

In Canada, herbal products for human use are known as natural health products (NHPs).

**Natural health products (NHPs).** This definition refers to a broad range of health products including traditional medicine and homeopathic medicine. NHPs refer to a substance or combination of substances from a plant or plant material, fungus, algae, bacterium, or non-human animal material, as well as an extract or a compound isolated from the above-mentioned products. It also includes mainly vitamins, a mineral, an amino acid, a probiotic, an essential fatty acid, traditional medicine, and homeopathic medicine as listed in the NHPR (schedule 1, subsection 1(1), included Natural Health Product Substances) [32]. These products are used and marketed for the purposes of treatment, mitigation or prevention of a disease, diagnosis, abnormal physical state in humans, disorder, modifying organic functions in humans to maintain or promote health, and restoring or correcting organic functions in humans [32].

Additionally, traditional medicine classified as an NHP must comply with the following definition: “medicine based on the sum total of knowledge, skills and practices based on theories, beliefs and experiences indigenous to different cultures, used in the maintenance of health, as well as in the prevention, diagnosis, improvement or treatment of physical and mental illness” [32].

## 6. United States of America

### 6.1. Native Americans in the United States of America and Traditional Medicine

The Indigenous people of the United States of America (USA) are mainly American Indians, and Alaskan and Hawaiian Natives with an estimated population of 5 million (1.5%) as per the last population census [34].

Health practices among Native Americans of the USA were practised for centuries before the arrival of the European colonisers and focused on the relationships between people (family, social group) and the environment during therapeutic sessions that can be supported by products such as herbs [35].

In particular, the American Indians and Alaskan Natives lost their lands and other resources during colonisation, impacting their way of life, including the suppression of their spiritual and healing practices that prevails to this day [35]. In 1787, Congress was granted broad constitutional power to negotiate with American Indians and Alaskan Natives, displacing them to reservations and separating them from their sacred sites, as well as medically important flora and fauna, leading to the loss of rights. In 1978, the USA Congress passed the American Indian Religious Freedom Act. However, many American Indians and Alaskan Natives are not federally recognised [35].

The practice of traditional Native American medicine lasted for centuries until the arrival of the European conquerors. However, this knowledge was devalued, and these practices were banned. Therefore, very little is known about these practices in human health, and archaeological remains and human remains are difficult to interpret. In particular, the study of the latter is limited due to the Native American Graves Protection and Repatriation Act enacted in 1990 [36]. However, the current tribal groups are trying to reconstruct these ancestral practices [35].

The study of Indigenous folk medicine based on wild plants in the USA is more than 100 years old [37]. It is estimated that 1100 plant species of the mesophytic forests of Appalachia have medicinal value [38]. These forests comprise eastern Tennessee, western North Carolina, and southeastern Virginia, which is a region with a diverse temperature range conducive to the growth of a great botanical variety.

### 6.2. Regulatory Status of Herbal Products (Botanical Products) in the United States of America

The Food and Drug Administration (FDA) is the protection agency of public health in the USA federal government that regulates the efficacy, safety, and security of human drugs, biological products, and food (dietary supplements, food additives, etc.), among others [39]. The FDA is part of the Department of Health and Human Services, which is administered by the United States Secretary of Health and Human Services. The FDA consists of nine organisations, including the Centre for Drug Evaluation and Research and the Centre for Food Safety and Applied Nutrition [40].

The USA does not have a national plan to integrate traditional and complementary medicine into conventional health services because it is regulated at the state level. However, it does have the National Centre for Complementary and Integrative Health to support research and provide information on complementary health products and practices [4] (Table 1).

Herbal products (botanical products) in the USA are regulated by the FDA according to intended use as: (a) foods; (b) dietary supplements under the Dietary Supplement Health and Education Act of 1994 [41]; (c) cosmetics; and (d) botanical drugs according to the Botanical Drug Development Guidance for Industry issued in 2016 [42].

In the USA, an herbal medicine is defined as “an herb is a plant or plant part used for its scent, flavour, or therapeutic properties” and that “are sold as powders, capsules, teas, extracts, and fresh or dried plants”. Thus, in the USA, an herbal medicine is considered a dietary supplement [41], while a botanical drug is an herbal medicine that has demonstrated efficacy and safety [42].

In this review we will only focus on botanical drugs and dietary supplements with herbal content.

#### 6.2.1. Botanical Drug

The FDA defines a botanical drug product as one that “contains materials of plant origin such as plant materials, macroscopic fungi, algae, or combinations thereof”. It must not contain animals or parts of animals unless it is a minor component in the botanical preparation. Botanical substances that are chemically modified or highly purified fermentation products are not considered botanical drug products. It also does not include highly purified substances from natural sources or chemically modified [42]. These products can be in the form of injections, tablets, powders, capsules, solutions, elixirs, or ointments [42]. In addition, if the botanical product is used “for the treatment, cure, diagnosis, mitigation or prevention of disease in humans”, it must be regulated as a drug. The development of a botanical drug should follow the recommendations included in the Botanical Drug Development Guidance for Industry [41], which is similar to chemically synthesised drugs. This guide describes the ways in which a botanical drug can be developed by industry for the purpose of marketing in the USA: (i) as an over-the-counter (OTC) drug monograph or (ii) as an approved new drug application (NDA) [42]. The differences are described below:

(A) “Marketing of botanical drugs as over-the-counter (OTC) drug monographs”. An OTC drug is one that is not considered a prescription drug, according to the United States Federal Food, Drug, and Cosmetic Act (FFDCA) section 503 (b) [43], which describes the conditions under which human drug products must have the supervision of a licensed pharmacist. Botanical drugs marketed as OTC may be eligible for appearance in the OTC Drug Monograph System and must be effective and safe according to the Code of Federal Regulations (CFR) (Title 21, part330, subpart B, sec 330.10) [44]. Additionally, a botanical drug substance can be included in the OTC Drug Monograph System and must be officially recognised by the United States Pharmacopeia and the National Formulary (USP-NF) drug monograph [45]. Currently, various botanical drug substances are in OTC drug review, such as psyllium and senna, which indicates that their safety and efficacy is still under evaluation. This process consists of three phases, requiring a federal register publication, to establish the standards for the marketing of OTC drugs (with monographs or non-monographs) under the OTC therapeutic drug category [42,43].
(B) “Marketing of botanical drugs under the new drug application (NDAs) scheme”: To be included in this scheme, it must be ruled out that there is no evidence of marketing in the USA or in another country for a botanical drug product, if the safety, efficacy, and therapeutic data are not sufficient to be registered as an OTC drug monograph, as well as if the proposed indication is not appropriate for non-prescription use. Under these conditions, the manufacturer must apply as an NDA to the FDA for its marketing as a botanical drug product with a proposed use. It could then obtain authorisation for prescription or OTC use, according to the characteristics and use indications of the product and whether it is safe to use without supervision of authorised personnel.

The application must include information on chemistry, manufacture, and product quality control; furthermore, the efficacy and safety must be proven in clinical studies [42]. Thus, FDA approval for the marketing of these products will be under FFDCA (201 and 505 sections) rules [43].

Quality controls are essential for botanical drug products because they have a heterogenous composition due to the fact that they come from nature. Therefore, it must be ensured that the beneficial effects are consistent across different batches of marketed products. Thus, it is desirable to consider raw material control, quality control, manufacturing control, biological assay, and clinical data to ensure therapeutic consistency [46]. It is important to mention that the United States Pharmacopeia (USP) organisation published a Compendium of Herbal Medicines; although its application is not mandatory, it is very useful.

#### 6.2.2. Botanical Drug Development in the Scheme of Investigational New Drug Applications (INDs)

In this group are products that do not have sufficient scientific information to be classified as NDA botanical drugs and therefore require further research. According to section 505 (i) of the FD&C Act and CFR part 312, if there is a need to obtain clinical data to support an NDA or an OTC monograph for the purpose of developing a botanical drug, the specifications contained in that act must be followed [43,44], and this will be referred to as investigational new drug applications (INDs).

In the INDs scheme, it is also relevant to include additional information besides the human risks, including identification of plant species, botanical raw material and analytical considerations, quality control tests of each batch, previous results, nonclinical pharmacology, nonclinical toxicology, and clinical pharmacology. In clinical studies for INDs (Phase 3), it is important to characterise the botanical drug product for the validity and reliability of the clinical information generated.

#### 6.2.3. Dietary Supplements

According to the Dietary Supplement Health and Education Act (DSHEA; 1994), dietary supplements are classified as a special food [41]. They cannot be presented as a dietary product or a food. A dietary supplement is defined as “a product that is taken by mouth and may include vitamins, minerals, herbs or other botanicals, amino acids, and substances such as enzymes, organ tissues, glandulars, and metabolites”. These can be marketed as “tablets, capsules, softgels, liquids or powders” [41]. They cannot be labelled to prevent, cure, mitigate, treat, or diagnose diseases. The labelling should include: “(1) the statement of identity (name of dietary supplement; (2) the net quantity of contents statement (amount of dietary supplement; (3) the nutrition labelling; (4) the ingredient list; (5) the name and place of business of the manufacturer, packer or distributor”. Furthermore, the product must comply with good manufacturing practices which are included in the Small Entity Compliance Guide: Current Good Manufacturing Practice in Manufacturing, Packaging, Labelling, or Holding Operations for Dietary Supplements [47]. In addition, amendments related to the labelling of dietary supplements are in the process of being approved. However, FDA approval is not required before marketing these products. The USP organisation also has a Dietary Supplements Compendium, although it is not mandatory.

Additionally, it is important to mention that the USP and the National Formulary (NF), known as USP-NF, contains standards for medicines, dosage forms, excipients, compounded preparations, and dietary supplements which are enforceable by the FDA for medicines manufactured and commercialised in the USA [43]. The USP is legally recognised in Section 503A of the 1997 Food Drug Administration Modernization Act and amended in 2013 by Congress [45].

## 7. Discussion

The use of herbal products has been very relevant and important worldwide and recognition of their contribution to human health has been increasing. However, these products, like any other medicinal products, can generate adverse effects as well as herb-drug interactions that can harm human health if there are no legal regulations for their manufacture and sale. In particular, the regulatory status of herbal products marketed under the USMCA agreement between Mexico, Canada, and the USA has notable differences.

Mexico has a long tradition in the use of herbal products and has nationally recognised traditional medicine in the General Health Law [4,18]. Canada also has both a national policy and a national program for T&CM but with regional implementation [4,30]. However, the USA does not have national policies for T&CM [Table 1].

In particular, Mexico has more specific regulations and classifies herbal products for human use into three groups: (a) herbal medicine; (b) herbal remedy; and (c) food supplement. These are under surveillance according to the General Health Law [18]. For example, herbal medicines as well as herbal remedies must comply with the Official Mexican Standards NOM-059-SSA1-2015 (good manufacturing practices for drugs) [48], NOM-248-SSA1-2011 (good manufacturing practices for drugs) [21], NOM-072-SSA1-2012 (labelling) [20], and NOM-073-SSA1-2015 (product stability) [49]. In particular, the herbal medicinal product needs to scientifically demonstrate therapeutic efficacy and safety. Herbal remedies do not require scientific demonstration of their therapeutic effects because their use is based on traditional or folk knowledge. For food supplements, registration is not required.

In Canada, products containing herbal components for human use are classified as NHPs, which include, for example, herbal medicines, dietary supplements, and traditional medicine, and are governed under the Food and Drug Act and Regulations [31,32]. NHPs must show safety and efficacy based on scientific data before they can be marketed.

The herbal products in the USA are regulated by the FDA and our focus is on dietary supplements and botanical drugs. The former is regulated through the Dietary Supplement Health and Education Act of 1994 [41] and is not subject to strict health surveillance. Nevertheless, they can cause harm to humans because it is not taken into account whether or not they contain herbal material and have not demonstrated efficacy and safety. It is therefore important that their consequences for health be scientifically investigated. Furthermore, as they are not considered drugs, they are not subject to the same requirements as drugs and do not have to be examined for safety and efficacy, which is the same situation as in Mexico for food supplements.

With regard to herbal medicines, so called in Mexico and Canada, and known as botanical drugs in the USA, scientific studies are required to guarantee their therapeutic efficacy and framework and application for their safety for human health.

In particular, in the USA, there is a very recent advance in the regulation of botanical drugs, which is supervised by the FDA according to the modifications made to the Botanical Drug Development Guidance for Industry issued in 2016 [42], which involve more specific recommendations and criteria to achieve registration of a botanical drug. In this sense, it is required to identify the active ingredients, mechanisms of action, safety, and efficacy for phases I, II, and III of clinical trials as well as to comply with good manufacturing practices in order to be placed in the USA market as a prescription drug. Moreover, botanical drugs that are good candidates are those herbal products with proven safety and efficacy and that have been used in a large-scale population that will enhance the efficacy success in phase II and III trials [42].

An important difference between a botanical drug in the USA and an herbal medicine in Mexico is the number of studies needed to prove the efficacy of the final product in the USA After approval by the FDA and registration, it is classified as a drug. For Mexico and the USA, an active ingredient isolated from plants is not a candidate to be considered as an herbal medicinal product [20,42].

The regulatory policies in the USA have been changing due to the authorisation of plant-based products in other regions of the world, which has also been growing in Mexico and Canada in recent years. At present, there are only two new botanical drugs legally authorised in the USA, namely veregen (sinecatechins) and fulyzaq (Crofelemer). Thus, the USA Centre for Drug Evaluation and Research has established that if it is not possible to fully characterise the active compounds of the botanical mixture, a chemical constituent profile, among others, can be selected [42]. Therefore, a chemical marker is necessary as required in Mexico according to the Herbal Pharmacopoeia.

In this regard, the USA has opened up great possibilities for the introduction of various herbal products as prescription drugs such as Cannabis-derived products. In Canada, a substance isolated from such products is also considered an herbal medicine if it complies with the legal framework [32]. In Mexico, a single active compound from plants is considered a prescription drug.

Other aspects are also relevant to ensure the quality of an herbal drug, such as the herbal raw material, methods for quality control across product batches, clinical data, and manufacturing control, among others, to achieve the expected therapeutic effects.

Thus, it is important to mention that several herbal products that are considered as herbal medicines in Mexico and Canada due to their scientific support, efficacy, and safety in humans are considered dietary supplements in the USA The latter do not require FDA approval prior to marketing, which does not ensure that they are free of ingredients or materials harmful to human health. In addition, compliance and responsibility for dietary supplements rests on manufacturers and distributors of these products. In Canada, botanical food supplements require authorisation prior to market introduction and must include information as if they were a drug (ingredients, non-medicinal ingredients, source, potency, strength, etc.) [32,33].

It is also relevant to mention that Mexico has one of the most complete indexes of Pharmacopoeias worldwide, including: (i) general Pharmacopoeia; (ii) the homeopathic pharmacopoeia; and (iii) the herbal pharmacopoeia, compared with Canada and the USA [50]. In the USA, the USP has a Compendium of Dietary Supplements that Mexico does not have.

The differences and similarities in the legal framework for herbal products in one of the world’s most important economic and trade zones [51] are related to their history, idiosyncrasies, and socio-economic and cultural aspects.

However, these differences in public policies related to herbal products in the North American region, as well as globally, affects the quality of these products. Moreover, evaluation of the efficacy and safety of herbal medicines or botanical drugs is more complex than that of conventional medicines because an herbal medicine or medicinal plant can contain several natural compounds or a mixture of different plants. This represents a challenge in controlling the raw material, which depend on environmental conditions, agricultural and harvesting practices, correct identification of herbal species, special cleaning methods, and good manufacturing practices among other relevant aspects. It is therefore important that the legal framework be amended in the future so that any plant-based product to be used for human health, including dietary supplements [52], is first evaluated and supported by scientific research to reduce health risks.

The main differences and similarities that can be highlighted in the regulatory framework between these three countries are partly related to the classification of herbal products and marketing criteria (Table 1 and Table 2). Thus, we can mention that the herbal medicines (Mexico), traditional medicine (Canada), or botanical drugs (USA) require documented efficacy, but in Mexico and Canada data from the literature is accepted [32]. Likewise, these products must comply with good manufacturing, labelling, and stability practices.

Mexico recognises herbal remedies, while Canada recognises traditional medicines, but the main difference between them is the documentation of the time of use of a given product, as well as its marketing. For example, in Canada, its use must be in the same dosage and presentation as recommended by traditional medicine. The USA does not integrate traditional or complementary medicine into conventional health services but due to the increased use of complementary and alternative medicine products, the FDA has developed a draft guidance and their regulation for industry [53].

In the case of dietary supplements (USA) and food supplements (Mexico), these products must comply with the labelling. Good manufacturing practices must be complied with for these products in the USA [41], while good food hygiene practices are required in Mexico. However, the particularity of these products is that in USA, if they voluntarily comply with the pharmacopoeia tests for dietary supplements, which do not exist in Mexico, they can be labelled with a USP verified mark, which indicates the approved quality and differentiates them from other dietary supplements.

Thus, it can be observed that there are risks for the existence of toxic components, or a risk of overdose for dietary supplements (USA) and food supplements (Mexico). It is also important that both producers and consumers have a responsibility when using OTC products, as their inappropriate use can also have health consequences. Therefore, it would be advisable to review their regulation and marketing requirements.

It is important to note that the regulation of herbal products marketed in the North American region will undergo modifications according to the needs that are detected because of trade and investments between the three nations that participate in the USMCA. Probably, the regulatory system of other countries with extensive experience in the use of these products can also serve as a model to improve the current regulations, so that it works better and meets the expectations of those who use them. It may be interesting to consider systems such as China, a country where traditional medicine originated approximately 3000 years ago [54]. In this country, registration processes have been established for products that have a long history of use in T&CM, and they are classified into innovative Chinese medicines, modified new Chinese medicines, generic Chinese medicines (same name and formulations), and classic Chinese medicine formulation (CCMF) [55]. Interestingly, the National Medical Products Administration (NMPA) in China has set to advance the regulatory capacity of traditional Chinese medicines (TCMs), including herbal medicines, with the adoption of Regulatory Science (RS), which is a comparative discipline that has been adopted by drug regulatory authorities in order to enhance the scientific rationale supporting their benefit/risk analysis and regulatory decisions based on the best available science [56]. In May 2019, the NMPA launched the Action Plan of Regulatory Science, which is the first initiative that harmonizes the global trend of RS development [57]. With the Action Plan of RS, the regulatory agency is committed to the development of new regulatory tools, standards, and methods, so that the regulatory decision-making process in the NMPA becomes more scientific, progressive, and adaptable in the new era of the regulatory paradigm. Therefore, the drug regulation system in China is expected to adhere to the five major attributes of innovation, quality, efficiency, system, and capacity. The Action Plan of RS covers ten key areas of SR development in China, impacting a wide range of regulated products, including the safety assessment of substances and prepared medicines (herbs, minerals, and animal substances) used in Traditional Chinese Medicine (TCM). The priority areas of the Action Plan are: (1) modernizing the regulatory system with a holistic approach; (2) advancing the methodology for the quality control of TCMs; (3) fostering the control mechanism of TCMs manufacturing process; (4) improving clinical evaluation of TCMs and leveraging real world data; (5) re-evaluation of TCM injection; (6) developing evaluation standards for classic TCM formula; (7) harnessing diverse data to improve pharmacovigilance of TCMs; (8) evaluating the value of integrative medicine in clinical practice with scientific research; (9) advancing the regulatory capacity to encourage innovation in TCMs; and (10) advancing a vision of collaboration for RS development in TCMs [58]. Thus, the development experiences of SR in TCM in China can become an important model for other countries interested in regulating all aspects related to herbal products used in T&CM to guarantee their quality. 

On the other hand, it is important to mention that due to the worldwide increase of herbal health products, the extinction of several plant species has been estimated. Therefore, it is important that when public policies are developed, they should be oriented towards the realisation of the common good through the fulfilment of duty. In this sense, the application of ethics in policy for the regulation of herbal products for sale should take into account the following relevant points based on an ethical framework for the provision of herbal medicines: (a) conditions must be in place so that rules and regulations can be enforced to regulate the production, use, and marketing of medicinal plants; (b) from the commercial aspect, it is important to develop species-priority-based production strategies due to the depletion of wild herbal resources; (c) from the social-ecological aspect, it is of great relevance to assess the performance of production systems and their relationship to livelihoods. However, it is unfortunate that only a proportion of medicinal plants have been adequately protected through nature reserves or botanical gardens. It is therefore important to develop strategies for plant conservation, cultivation practices, good agricultural practice, and sustainable use.

It is also important to mention that in herbal medicine, there are four values to be applied: (i) care for well-being of the people, communities, and environments from which the herbs are obtained; (ii) respect in accepting different preferences, customs, and cultures; (iii) honesty; and (iv) equity in distribution and availability of treatments of the same quality to all people, fair charging of products, and accountability.

## 8. Conclusions

The use of herbal products as a therapeutic alternative for primary human health care has increased in the North American region mainly due to the increase in life expectancy and low cost. This is due to the impetus that the WHO has given at the global level for the use of traditional and complementary medicine. Differences in the legal framework between Mexico-Canada-USA for these products are related to their history, idiosyncrasies, and socio-economic and cultural aspects, but human health is the priority. However, efforts to reduce health risks need to be intensified through close health surveillance and monitoring. Furthermore, more regular evaluations of legislation, considering the experience of producers, consumers, and the scientific community, are also important. This is in order to reduce the contamination of products that do not require specific standards for their marketing, e.g., support with scientific data or monitoring in their manufacture because they impact human health. Close surveillance and health monitoring is also important, as well as our responsibility as consumers to report any adverse effects.

It is very important that both producers and consumers have a social and ethical responsibility in the sale and consumption of OTC products, as they can cause damage to health if not used correctly, in addition to the contamination that these products may have during their manufacturing if they are not closely monitored. Therefore, a social commitment is needed for the most vulnerable population.

Although there are differences in the legal framework in this region, human health is prioritised and protected. It is therefore necessary to step up efforts to reduce the health risks from the use of these products by carrying out more regular assessments of the legislation, with the experience of producers, consumers, and the scientific community.

## Figures and Tables

**Table 1:** Regulatory status of herbal products in the North American region.

Classification	Mexico	Canada	USA
National policies on T&CM	YES	YES	NO
Laws or regulations at the national or state level for T&CM	YES	YES	YES
National program on T&CM	YES	YES	data not available
National office for T&CM	YES	YES	YES
National committee of experts for T&CM	YES	YES	YES
National research institute for T&CM	NO	NO	YES
Regulation of herbal medicines	YES	YES	YES
Registration system for herbal medicines	YES	YES	YES
Population using T&CM (including indigenous medicine)	YES	YES	YES

Data Source, World Health Organization 2019 [4]; T&CM, Traditional and complementary medicine.

**Table 2:** Comparison of herbal products in North America.

	Herbal Products	Herbal Pharmacopoeia	Herbal Medicine
Mexico	Herbal medicines, herbal remedies, food supplements each have a different definition	Mexican Herbal Pharmacopoeia	Herbal medicine requires scientific studies
Canada	Herbal medicines, herbal remedies, dietary supplements, food supplements are considered as natural health products	No pharmacopoeia of its own relies on others	Herbal medicine requires scientific studies
U.S.A	Foods, dietary supplements, botanical drugs each have a different definition	National formulary (USP-NF)	Botanical drug requires scientific studies

## Data Availability

Not applicable.
